# Prognostic significance of histological grade in low-risk endometrial cancer

**DOI:** 10.7150/ijms.77152

**Published:** 2022-10-24

**Authors:** Seong Eun Bak, Ji Geun Yoo, Sung Jong Lee, Joo Hee Yoon, Dong Choon Park, Sang Il Kim

**Affiliations:** 1 Department of Obstetrics and Gynecology, Seoul St. Mary's Hospital, College of Medicine, The Catholic University of Korea, Seoul, Republic of Korea.; 2 Department of Obstetrics and Gynecology, Daejeon St. Mary's Hospital, College of Medicine, The Catholic University of Korea, Seoul, Republic of Korea.; 3 Department of Obstetrics and Gynecology, St. Vincent's Hospital, College of Medicine, The Catholic University of Korea, Seoul, Republic of Korea.

**Keywords:** endometrial cancer, uterine cancer, low risk, risk factors, histological grade

## Abstract

**Objective:** Investigate the risk factors for recurrence in patients with low-risk endometrial cancer.

**Method:** A retrospective review was performed to identify patients who underwent primary surgical treatment for endometrial cancer from December 2009 to December 2020. Patients who met the following criteria were included in the study: (a) International Federation of Gynecology and Obstetrics stage IA, (b) endometrioid-type histology, (c) histological grade 1 or 2.

Univariate and multivariate analyses using Cox proportional hazards model to evaluate effects of prognostic factors. Disease-free survival and overall survival were calculated using the Kaplan-Meier method.

**Results:** A total of 171 patients with low-risk endometrial cancer were included in the study. Recurrence was detected in 9 patients. Histological grade was found to be independent risk factors for recurrence in women with low-risk endometrial cancer (OR 8.255, 95% confidence interval (CI) 1.585 - 42.981; p = 0.012).

**Conclusion:** The results of this study suggest that grade 2 disease should be considered a significant prognostic factor for the recurrence of low-risk endometrial cancer.

## Introduction

Endometrial cancer (EC) is the most common gynecologic malignancy in developed countries 1. Approximately 65,950 new cases of EC and 12,550 deaths related to EC are expected to occur in the United States in 2022 2. The primary treatment for EC is surgery, and the standard staging surgery is total hysterectomy and bilateral salpingo-oophorectomy with lymph node assessment 3. Adjuvant treatment is recommended for EC based on the estimated risk for recurrence 4.

Most patients with EC are diagnosed with uterine-confined, stage I disease 5. Stage I EC can be subdivided into low-, intermediate-, and high-risk disease based on the following risk factors: histological subtype, grade, depth of myometrial invasion (MMI), lymphovascular space invasion (LVSI), and tumor diameter 6. Low-risk EC is defined as stage I disease with an endometrioid histological subtype (grade 1 or 2) and less than 50% MMI 5. It is generally accepted that adjuvant treatment is not necessary for low-risk EC because its recurrence rates are low 7. However, 3%-10% of patients with low-risk EC experience relapse 8. Moreover, the risk factors for recurrence of low-risk EC have not been clearly identified. The identification of patients with low-risk EC who have an elevated risk for relapse is critical for the development of individualized and tailored postoperative treatment regimens. Therefore, the aim of this study was to evaluate the impact of the risk factors for recurrence in patients with low-risk EC.

## Materials and Methods

This retrospective study was approved by the Institutional Review Board of the Catholic University of Korea. The requirement for informed consent was waived owing to the retrospective nature of the study. The study was conducted in accordance with the principles of the Declaration of Helsinki.

We reviewed the cancer registry of our institution and identified patients who underwent primary surgical treatment for EC from December 2009 to December 2020. The inclusion criteria were as follows: patients with International Federation of Gynecology and Obstetrics stage IA EC, and patients who had tumors with an endometrioid histological subtype (grade 1 or 2). Patients with incomplete clinicopathological or follow-up data were excluded. All patients underwent standard staging surgery. In addition, all patients received postoperative adjuvant radiation therapy (RT) based on adverse risk factors.

Disease-free survival (DFS) was defined as the duration from the date of primary surgery to the date of the first disease recurrence. Recurrence was assessed using biopsy or imaging studies. If recurrence did not occur, observation was censored at the date of death or the last follow-up. Overall survival (OS) was defined as the duration from the date of primary surgery to the date of cancer-related death or the last follow-up. DFS and OS were analyzed using the Kaplan-Meier method, and the curves were compared using the log rank test. Univariate and multivariate analyses using Cox proportional hazards model to evaluate effects of prognostic factors. All statistical analyses were performed using Statistical Package for the Social Science (SPSS) software, version 22.0 (SPSS Inc., Chicago, IL, USA). *P* < 0.05 was considered statistically significant.

## Results

We identified and included 171 eligible patients according to the inclusion and exclusion criteria of this study. Table 1 shows the clinical and pathological demographic characteristics of the patients with low-risk EC according to recurrence status. A total of nine (5.3%) recurrences were observed. Patients who showed recurrence were more likely to have grade 2 disease that those who did not show recurrence (p=0.004). Other factors, including age, body mass index, menopause status, tumor size, and LVSI status, were not significantly different between the two groups. In addition, the rates of adjuvant RT in both groups were similar (14.8% vs 11.1%, p=0.759).

Risk factors for recurrence are shown in table 2. Univariate analysis revealed that patients with grade 2 disease showed significantly decreased DFS (p=0.016). Age, menopause status, tumor size, LVSI status, lymph node dissection, and adjuvant RT were not associated with DFS. However, grade 2 disease was an independent prognostic factor for DFS in the multivariate analysis. Regarding OS, none of the above-mentioned factors showed prognostic significance for OS in the univariate and multivariate analyses.

**Figure 1:**
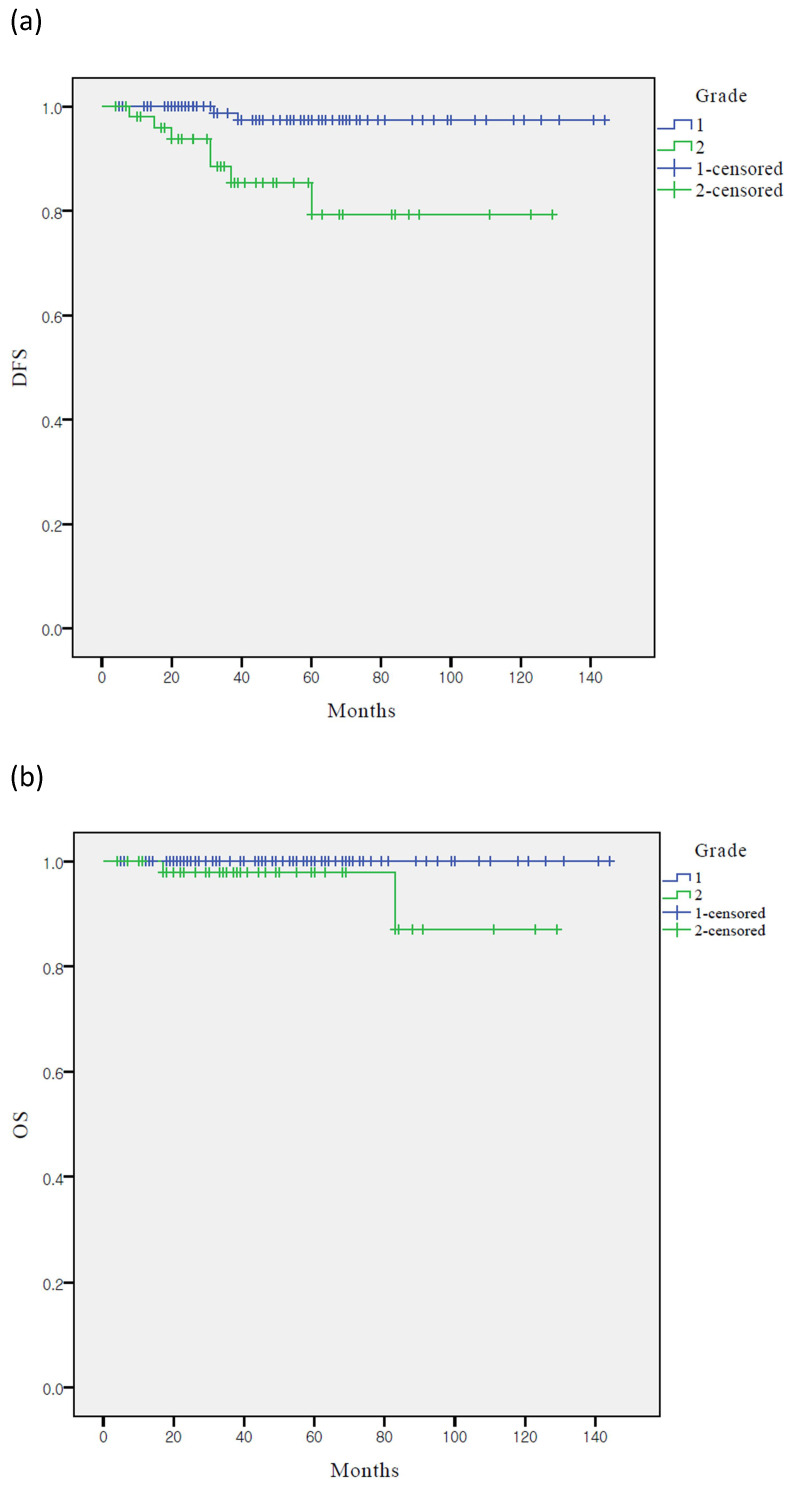
Survival curves according to grade: (**a**) Kaplan-Meier survival curves for DFS (**b**) Kaplan-Meier survival curves for OS. DFS, disease-free survival; OS, overall survival

The Kaplan-Meier analysis showed that the five-year DFS rates in the grades 1 and 2 groups were 97.5% and 79.3% (*p* < 0.001), respectively, whereas the five-year OS rates were 98.9% and 98.0%, respectively (*p* = 0.222) (Figure 1). Only DFS was significantly lower in the grade 2 group than in the grade 1 group. However, the OS of the two groups were not significantly different.

## Discussion

The survival of rate of EC is generally good. It has been reported that the five-year survival rate for patients with surgical stage I disease, without any adverse risk factors other than grade and MMI, is 92.7% 9. Multiple adverse risk factors of EC, including histological type, grade, depth of MMI, and LVSI, have been defined 6. However, the risk factors for the recurrence of low-risk EC are unclear.

The incidence of EC is increasing rapidly worldwide. Low-risk EC accounts for approximately 60-70% of all cases of EC grossly confined to the uterus 10. Although the recurrence rate of low-risk EC is very low, the absolute number of recurrences is considerable. Thus, identification of subgroups of women with EC who have an increased risk for relapse is critical. The findings of the present study indicate that grade 2 disease is a significant prognostic factor for recurrence in patients with low-risk EC. In addition, the results show that patients with grade 2 EC are eight times more likely to show recurrence than those with grade 1 EC.

Several phase III trials have been conducted to assess the use of adjuvant RT for the treatment of uterine-confined EC 7, 11. Adjuvant RT improves DFS in patients with selected risk factors. However, postoperative radiotherapy is generally not indicated for patients with low-risk EC. The National Comprehensive Cancer Network guidelines recommend observation after surgery for low-risk EC. However, adjuvant vaginal brachytherapy is suggested in cases of LVSI and/or for patients older than 60 years 3.

Grade has been identified as an independent predictor of recurrence in EC. Mariani et al. reported that grade 3 disease is the strongest predictor of vaginal relapse 12. In addition, the results of the multivariate analysis performed in the PORTEC 1 trial confirmed the prognostic significance of grade 3 disease for locoregional recurrence 13. On the other hand, a study on the comparison of vaginal brachytherapy versus observation for the management of stage IA grades 1 and 2 EC showed similar overall recurrence and survival rates for the two grades 14. As a result, stage IA disease with an endometrioid histological subtype (grade 1 or 2) was defined as low-risk EC 15. However, the results of the present study indicate that grade 1 and grade 2 low-risk EC must be discriminated. The results showed that patients with grade 2 low-risk EC are eight times more likely to show recurrence than patients with grade 1 disease. Thus, in addition to patients with LVSI and/or patients older than 60 years, patients with grade 2 disease can be candidates for adjuvant RT.

This study had several limitations. First, owing to the retrospective nature of the study, the possibility of inherent bias cannot be excluded. Second, the number of enrolled patients may have been insufficient. Thus, the findings of this study need to be confirmed in future studies with larger cohorts. Third, centralized pathologic review was not performed in this study. Forth, molecular analysis is not included.

In conclusion, the results of this study suggest that grade 2 disease should be considered a significant prognostic factor for the recurrence of low-risk EC. However, further prospective studies are needed to confirm this finding.

## Data Availability

The data that support the findings of this study are available on request from the corresponding author.

## Figures and Tables

**Table 1:** Clinical and pathological characteristics of patients (n=171).

	No recurrence (n = 162, %)	Recurrence (n = 9, %)	*p* value
Age (years), median (range)	54 (30 - 81)	53 (37 - 74)	0.538
BMI (kg/m^2^), median (range)	24.9 (15.2 - 39.3)	25.9 (21.5 - 29.7)	0.114
Menopause			0.490
No	60 (37.0)	2 (22.2)	
Yes	102 (63.0)	7 (77.8)	
Grade			0.004^*^
1	116 (71.6)	2 (22.2)	
2	46 (28.4)	7 (77.8)	
Tumor size (cm), median (range)	2.0 (0 - 9.8)	2.5 (0.8 - 5.0)	0.436
LVSI			0.426
Absent	153 (94.4)	8 (88.9)	
Positive	9 (5.6)	1 (11.1)	
LN assessment			0.739
No LND	27 (16.7)	0	
PLND only	36 (22.2)	3 (33.3)	
PLND & PALND	99 (61.1)	6 (66.7)	
Adjuvant RT			0.759
No	138 (85.2)	8 (88.9)	
Yes	24 (14.8)	1 (11.1)	
Median follow-up (months)	49 (12 - 144)	41 (17 - 83)	0.698

BMI, body mass index; LVSI, lymphovascular space invasion; LN, lymph nodes; LND, lymphadenectomy; PLND, pelvic lymphadenectomy; PALND, para-aortic lymphadenectomy; RT, radiotherapy.

**Table 2:** Univariate and multivariate analysis of prognostic factors for disease-free survival (n = 171).

Characteristics	Univariate analysis			Multivariate analysis		
	OR	95% CI	*p value*	OR	95% CI	*p value*
Age						
< 60	1 (Ref)	-	-			
≥ 60	0.474	0.079 - 2.841	0.414			
Menopause						
No	1 (Ref)	-	-			
Yes	2.500	0.432 - 14.475	0.306			
Grade						
1	1 (Ref)	-	-	1 (Ref)	-	-
2	8.193	1.492 - 44.989	0.016^*^	8.255	1.585 - 42.981	0.012^*^
Tumor size						
< 2cm	1 (Ref)	-	-			
≥ 2cm	1.701	0.343 - 8.432	0.516			
LVSI						
Absent	1 (Ref)	-	-			
Positive	2.814	0.090 - 88.235	0.556			
LND						
No	1 (Ref)	-	-			
Yes	2.050	0.594 - 12.848	0.406			
Adjuvant RT						
No	1 (Ref)	-	-			
Yes	0.213	0.007 - 6.654	0.378			

Covariates with *p* < 0.05 on univariate analysis were included in multivariate model. OR, odds ratio; CI, confidence interval; Ref, reference; LVSI, lymphovascular space invasion; LND, lymphadenectomy; RT, radiotherapy.
